# Incorporating development of a patient-reported outcome instrument in a clinical drug development program: examples from a heart failure program

**DOI:** 10.1186/s12955-016-0529-0

**Published:** 2016-09-15

**Authors:** Ingela Wiklund, Milena Anatchkova, Hafiz Oko-Osi, Robyn von Maltzahn, Dina Chau, Fady I. Malik, Donald L. Patrick, John Spertus, John R. Teerlink

**Affiliations:** 1 Evidera, Metro Building 6th Floor, 1 Butterwick, London, W6 8DL UK; 2 Evidera, Bethesda, MD USA; 3 Amgen Inc., Thousand Oaks, USA; 4 Cytokinetics, Inc., San Francisco, CA USA; 5 University of Washington, Seattle, WA USA; 6 University of Missouri, Kansas City, KS USA; 7 San Francisco Veterans Affairs Medical Center and University of California San Francisco School of Medicine, San Francisco, CA USA

**Keywords:** Patient-reported outcome, Qualitative research, Heart failure, FDA guidance, Instrument development, Omecamtiv mecarbil

## Abstract

**Background:**

Patient-reported outcome (PRO) measures can be used to support label claims if they adhere to US Food & Drug Administration guidance. The process of developing a new PRO measure is expensive and time-consuming. We report the results of qualitative studies to develop new PRO measures for use in clinical trials of omecamtiv mecarbil (a selective, small molecule activator of cardiac myosin) for patients with heart failure (HF), as well as the lessons learned from the development process.

**Methods:**

Concept elicitation focus groups and individual interviews were conducted with patients with HF to identify concepts for the instrument. Cognitive interviews with HF patients were used to confirm that no essential concepts were missing and to assess patient comprehension of the instrument and items.

**Results:**

During concept elicitation, the most frequently reported HF symptoms were shortness of breath, tiredness, fluid retention, fatigue, dizziness/light-headedness, swelling, weight fluctuation, and trouble sleeping. Two measures were developed based on the concepts: the Heart Failure Symptom Diary (HF-SD) and the Heart Failure Impact Scale (HFIS). Findings from cognitive interviews suggested that the items in the HF-SD and HFIS were relevant and well understood by patients. Multiple iterations of concept elicitation and cognitive interviews were needed based on FDA request for a broader patient population in the qualitative study. Lessons learned from the omecamtiv mecarbil PRO/clinical development program are discussed, including challenges of qualitative studies, patient recruitment, expected and actual timelines, cost, and engagement with various stakeholders.

**Conclusion:**

Development of a new PRO measure to support a label claim requires significant investment and early planning, as demonstrated by the omecamtiv mecarbil program.

## Background

In a patient-centered healthcare system, incorporating the patient’s perspective in research has become a priority [1, 2]. Patient-reported outcome (PRO) data may be used to support label claims for promotional purposes and/or scientific dissemination under specific guidance from the US Food and Drug Administration (FDA) [3, 4] and in general by the European Medicines Agency (EMA) [5]. The specific guidance from the FDA was published in 2009 [3], and the FDA’s Patient-Focused Drug Development Initiative was developed in 2013 [4]. Pharmaceutical companies may use PRO information to distinguish a product from a competing product, and to contribute to discussions with Health Technology Assessment (HTA) agencies and payers to support reimbursement decisions [6, 7]. An important aspect of a clinical program is to use tools that enable patients’ symptoms and symptom impacts on their lives to be captured and quantified. PROs are therefore essential in providing a metric by which treatment success can be measured.

Current regulatory requirements for PRO measures to be used in clinical trials are not consistent between regulatory agencies. The FDA has issued the most specific and stringent guidance to be considered in the development of PRO measures intended for use in label claims [3], while the EMA has published a reflection paper on the use of health-related quality of life measures in drug evaluations [5]. To support a labeling claim in the US, a PRO endpoint must be at least a pre-specified key secondary endpoint [3], whereas the EMA suggests that the PRO endpoint be driven by an a priori formulated hypothesis and detailed in the statistical analysis plan of a clinical trial that is suitably powered for the PRO analysis [5]. Because of these differences, sponsors must take differing regional agency requirements into consideration in global development programs.

An important component of the FDA guidelines is that a PRO measure must demonstrate content validity, which is the extent to which the instrument measures the concept of interest. In addition, the FDA specifies that a PRO instrument needs to be well defined, reliable, used in accordance with the instrument’s documented measurement capability, and to have demonstrated validity in the target patient population [3]. Several descriptions of the process recommended in the development of a good quality PRO measure have been published [8–11], but reports of the practical considerations of integrating development of a PRO measure into a clinical program are rare.

The importance of integrating a PRO measure in randomized clinical trials (RCTs) of drugs for the treatment of cardiovascular disease (CVD) has recently been highlighted [6]. It is important that patients are able to describe the impact of a novel treatment on their symptoms, functions, and health-related quality of life using scientifically sound PRO measures in CVD RCTs. Since the release of the FDA PRO guidance, no sponsor has achieved a PRO label claim for the treatment of chronic HF (CHF), even though old label claims for carvedilol and other products exist. PROs developed prior to the guidance may have been acceptable as label claims in the past. However, a recent literature review of PROs for chronic HF patients concluded that none of the existing disease-specific PRO instruments for HF satisfy all criteria outlined in the current FDA guidance [12], and therefore may no longer be considered adequate to support label claims.

The goal of this paper is to present the steps for integrating the development of PRO measures into a clinical drug development program with the aim of achieving a label claim with the FDA on the severity of symptoms and their impact on functioning for patients with HF. We will provide real-world examples of the processes, challenges, solutions, and lessons learned as part of the development of a symptom and impact measure integrated into a HF clinical trial program for omecamtiv mecarbil. Omecamtiv mecarbil is a selective, small molecule activator of cardiac myosin [13] that has been demonstrated to increase cardiac performance in animal models [14], healthy human volunteers [15], and patients with CHF [16].

### Process required for pro development: a real-world example

**Fig. 1:**
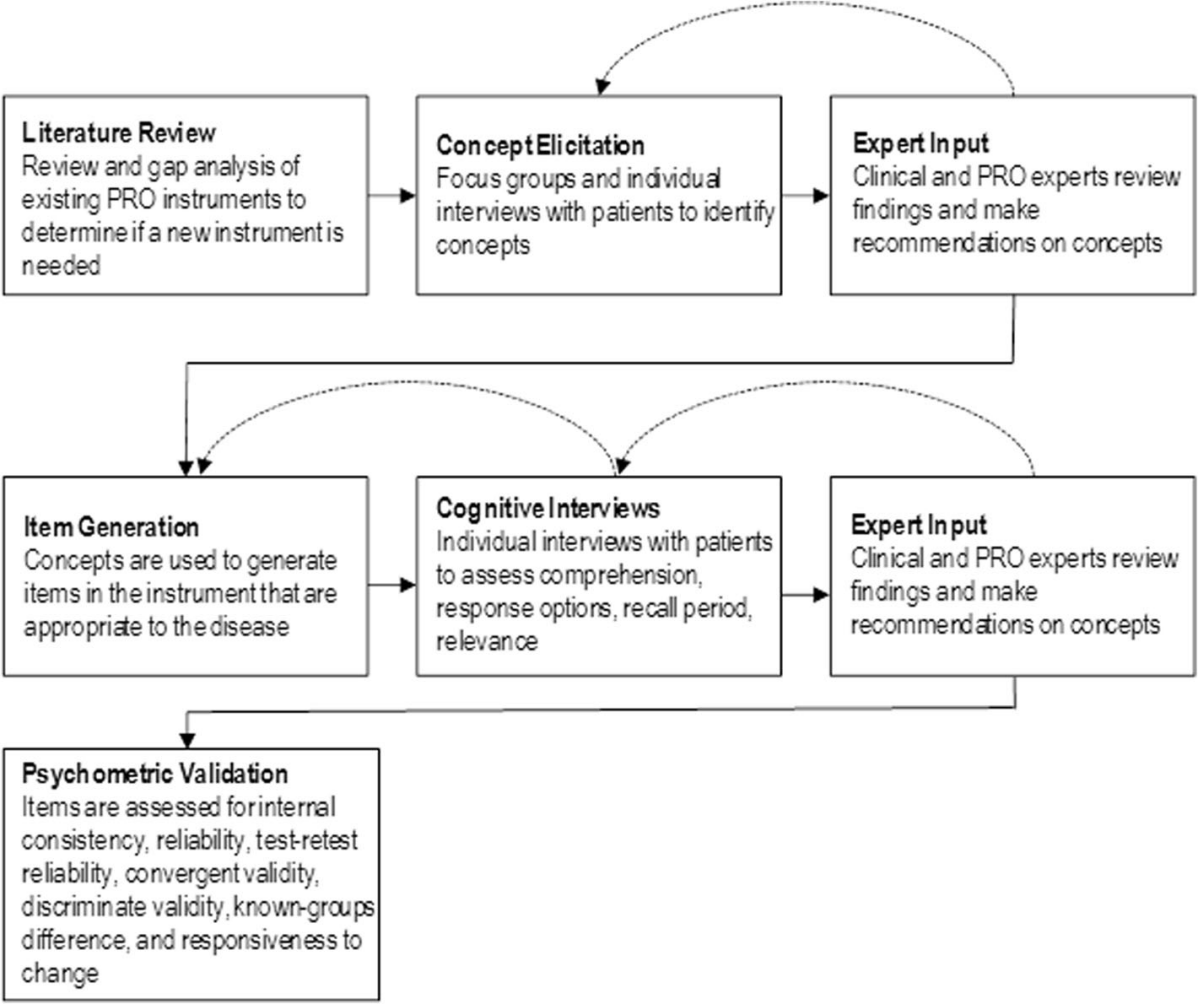
Key steps in the process of developing a PRO measure. Multiple iterations of concept elicitation, item generation, and cognitive interviews may be required (dashed arrows)

PROs can extend the insights of a product in development beyond mortality and morbidity benefits, by describing the impact of treatment from the patient’s perspective. Development of a PRO strategy must be considered early in a product’s clinical development program and a decision to develop a new PRO measure needs to be made as early as when phase 1 clinical studies are conducted. Before a decision to develop a new PRO instrument can be made, information such as past PRO labels and PRO development activities of products in the same therapeutic area, and the incremental commercial value of PROs are often considered. This defines the first step in PRO development, which is to conduct an extensive literature review to survey existing instruments, including a review of psychometric characteristics (reliability, validity, ability to detect change) of these measures and whether a label claim based on the measures has been successful (Fig. 1). A gap analysis of existing instruments will identify the extent to which they meet FDA PRO guidance criteria. Three possible outcomes of the literature review are: identification of a suitable PRO measure; identification of a measure that only needs modification; or a need to develop a new PRO measure. If the decision is made to develop a new PRO measure, a PRO committee comprising clinical experts in the therapeutic area of interest and PRO development experts should be formed.

*Omecamtiv mecarbil experience: A literature review and gap analysis* [12] *was conducted after completion of phase I and phase IIa studies of omecamtiv mecarbil* [15, 16]*. The results of the review identified multiple PRO measures for HF symptoms and impacts, but none was considered to meet fully the criteria put forward by the FDA in the PRO guidance document for use in a clinical trial to support a label claim, particularly due to gaps in documentation of content validity* [3]*. The FDA is most favorable of direct measures of symptoms and physical limitations as opposed to more abstract concepts of social limitations and patients’ perceptions of their quality of life* [3]*. Therefore, a decision was made to develop two new measures: one to assess the symptoms of CHF and one to assess the impact of symptoms on functioning. A PRO development committee of clinical and PRO experts was formed.*

*Lessons learned: The number of existing PRO measures and the extensive use of existing PRO measures do not guarantee that any existing measure meets current FDA PRO guidance criteria* [12]*.*

If a suitable measure is not found, the next step is to conduct qualitative research to identify concepts for the instrument and document content validity. Content validity shows that each of the items/domains of an instrument is appropriate and comprehensive relative to its intended measurement concept, population, and use [3]. Concept elicitation is a process that has been used to identify key concepts related to an individual patient’s symptoms and functioning with respect to the disease and treatment, with an effort to capture the patients’ experiences using their own words [10]. Concept elicitation can be based on focus groups or individual interviews, or preferably using both. Recruitment of a representative population in terms of demographic and clinical characteristics of the target population (including gender, ethnicity, educational level, symptom severity, comorbid conditions, etc.) represent important aspects to be taken into account. Concept elicitation is considered to be complete when saturation of concepts is achieved, defined as the point at which no substantially new themes, descriptions of a concept, or terms are introduced with additional interviews [17]. When concept elicitation is completed, the PRO instrument is drafted.

*Omecamtiv mecarbil experience: An institutional review board (IRB)-approved qualitative study was conducted; patients provided written informed consent before initiation of study-related activities. One-on-one concept elicitation interviews and focus groups were conducted by trained interviewers and moderators using semi-structured interview guides. Each session was recorded and subsequently transcribed for analysis using qualitative analysis software. An open coding approach was used in the analysis. Additional concept elicitation interviews were conducted until saturation was achieved.*

*Three concept elicitation focus groups and six individual interviews were conducted with 19 US adults with CHF (mean age 73.8 [standard deviation (SD) = 13] years; 21 % female; 79 % white; 11 % New York Heart Association [NYHA] class IV disease). The most frequently reported symptoms due to HF in concept elicitation focus groups and interviews were shortness of breath (reported by 74 % of patients), tiredness (58 %), fluid retention (58 %), fatigue (47 %), dizziness/light-headedness (32 %) swelling (32 %), weight fluctuation (26 %), and trouble sleeping (21 %). Other symptoms mentioned by less than 20 % of participants included difficulty concentrating, muscle problems (pain, cramps, numbness), loss of appetite, and bloating. Concept saturation of symptoms was reached by the end of the interviews.*

*HF had the greatest negative impact on physical (difficulty walking [58 %], needing frequent rests [54 %], reduced ability to do household chores [37 %]), social (reduced social/family interactions [37 %], participation in recreational activities/hobbies [26 %]), and emotional (frustration stemming from diminished ability or inability to complete activities [26 %]) domains. Other impacts on functioning mentioned by less than 20 % of participants included difficulty carrying/lifting objects, running, feeling dependent on others, dressing, impacts on intimate relationships, depression, and mood swings. Concept saturation of impacts was reached by the end of the interviews.*

*Initial item pools of two HF measures, the Heart Failure Symptom Diary (HF-SD) and Heart Failure Impact Scale (HFIS), were created based on the results of the concept elicitation focus groups and interviews, along with experts, and input from PRO, translation, and clinical experts, and a review of available literature. All items were programmed in an electronic device for further testing.*

*Lessons learned: A recruitment strategy to ensure inclusion of patient groups that are difficult to recruit (e.g., female patients with CHF and patients with severe [NYHA class IV] disease) is an important consideration at the early stages of development, but can present practical challenges by slowing the development process. Use of a recruitment tracker and continuous contact with study sites to understand which patients are available are helpful strategies, as is securing back-up sites. Close relationships with hospital settings may enhance recruitment of patients with acute and severe disease.*

After drafting an initial item pool based on the results of concept elicitation, cognitive interviews are conducted to confirm that no important concepts are missing in the draft instrument and that the respondents understand the wording and intended meaning of the items. Results from the cognitive interviews are used to revise the instrument. The purpose of cognitive interviews is to assess the patient’s comprehension of the instrument and the items and response options relative to their intended meaning and to assess the comprehensiveness of content to ensure that no important items are missing that would influence evaluation of the targeted concept [11].

*Omecamtiv mecarbil experience: Cognitive interviews using the HF-SD and HFIS electronic PRO (ePRO) measures were conducted with 18 adult US patients with CHF (mean age 68.8 [SD = 12] years, 33 % female, 83 % white, 22 % NYHA class IV disease). Findings from the cognitive interviews suggested that items of the HF-SD and HFIS were relevant and well understood by patients with CHF. Response options were also well understood and appropriate for the items. The recall periods for the HF-SD and HFIS were adequate for recollection of symptom experience and impacts due to HF. Results from cognitive interviews also suggested the need for two additional items of impacted activities: indoor and outdoor activities were changed to separate items, and lifting and carrying were changed to separate items. Some items were reworded for clarity.*

*Lessons learned: Achieving saturation of concepts in the concept elicitation focus groups and interviews ensured that no new concepts were found in the cognitive interviews, and only the separation of related items was needed for the HF-SD and HFIS following the cognitive interviews.*

A key feature of PRO development is the iterative nature of the process. Multiple waves of concept elicitation and cognitive interviews may be required to achieve saturation and sufficient evidence of content validity. Early engagement of the FDA in the PRO development process is important to ensure that the evidence for content validity is considered to be acceptable.

*Omecamtiv mecarbil experience: Results of concept elicitation interviews and cognitive interviews were presented to the FDA with request for feedback on the presented evidence in support of the content validity of the two measures. A second round of concept elicitation interviews and cognitive interviews was suggested by the FDA to solicit input from patients with specific demographic (women, African Americans) and clinical (NYHA class IV disease) characteristics. Additional concept elicitation interviews were therefore conducted with 15 US adults with CHF (mean age 59.7 [SD = 10.8] years; 73 % female; 47 % white; 13 % NYHA class IV disease). No participants were included in both rounds of qualitative interviews. The second round of concept elicitation interviews supported the concepts endorsed in the first round of interviews, no new concepts were identified, and no changes were made to the instruments. Additional cognitive interviews were conducted with ten additional US adults with CHF (mean age 68.6 [SD = 9] years; 30 % female; 40 % white; 20 % NYHA class IV disease). The findings from the additional cognitive interviews demonstrated that items of the HF-SD and the HFIS were well understood and relevant to CHF patients, supporting the findings of the initial cognitive interviews.*

*Lessons learned: A challenge was the recruitment of patients with NYHA class IV disease outside of large, specialized academic centers, as current treatment regimens are effective and prevent development of HF to this stage in many patients. While theoretically important, the successful saturation of concepts in the initial round of qualitative studies meant that no new concepts were identified in the second round.*

The final step in the development of a PRO measure is to perform a quantitative study to identify the final set of items, to further document content validity, and to assess the measurement properties. The PRO can be implemented in a proof-of-concept study or in a stand-alone study for validation purposes [3]. Stand-alone studies (which can also be run in parallel with a phase 2 trial) are designed to focus on assessment of the measurement properties of the instrument; however, they are costly, increase timelines, and do not provide information on effects of treatment. There are, however, situations where a stand-alone trial may be necessary, such as when a highly effective treatment provides rapid improvement, and there may not be a sufficient number of stable subjects between study visits, or when the decision to develop a PRO is made after the phase 2 study has already been started. The incorporation of PRO testing in a phase 2 clinical trial is cost- and time-efficient, and provides information on the drug being tested as an added benefit. Altogether these aspects represent advantages compared to a stand-alone study. Additional considerations for use in a clinical trial are the mode of administration (e.g., ePRO vs pencil and paper), and translations and cross-cultural issues for global trials. Without evidence of validity, reliability, responsiveness, and interpretability of an instrument prior to its use in pivotal trials, results will likely not be acceptable to regulatory bodies.

*Omecamtiv mecarbil experience: The HF-SD and HFIS measures were piloted in a phase 2 trial to assess item performance in order to identify and select the final instrument items. This trial also provided the opportunity for an initial assessment of the psychometric properties of the final measures. The measures were administered as ePRO assessments, which were initially tested as part of the cognitive interviews, and enabled an assessment of the usability of electronic data capture in HF patients in advance of proceeding with the electronic mode of administration in the phase 3 clinical trial. In addition to providing initial evidence of the measurement properties of the HF-SD and the HFIS, this step provides additional evidence for the measure’s content validity.*

*Lessons learned: Including ePRO administration in the cognitive interview phase provided information on usability and avoided a study to assess concordance between paper and ePRO modes of administration, which is time- and cost-consuming. Testing for feasibility and ease of use of ePRO administration early in development is useful before embarking on its use in clinical trials.*

### Practical and logistical issues: key learnings from the omecamtiv mecarbil clinical program

#### Timelines

**Fig. 2:**
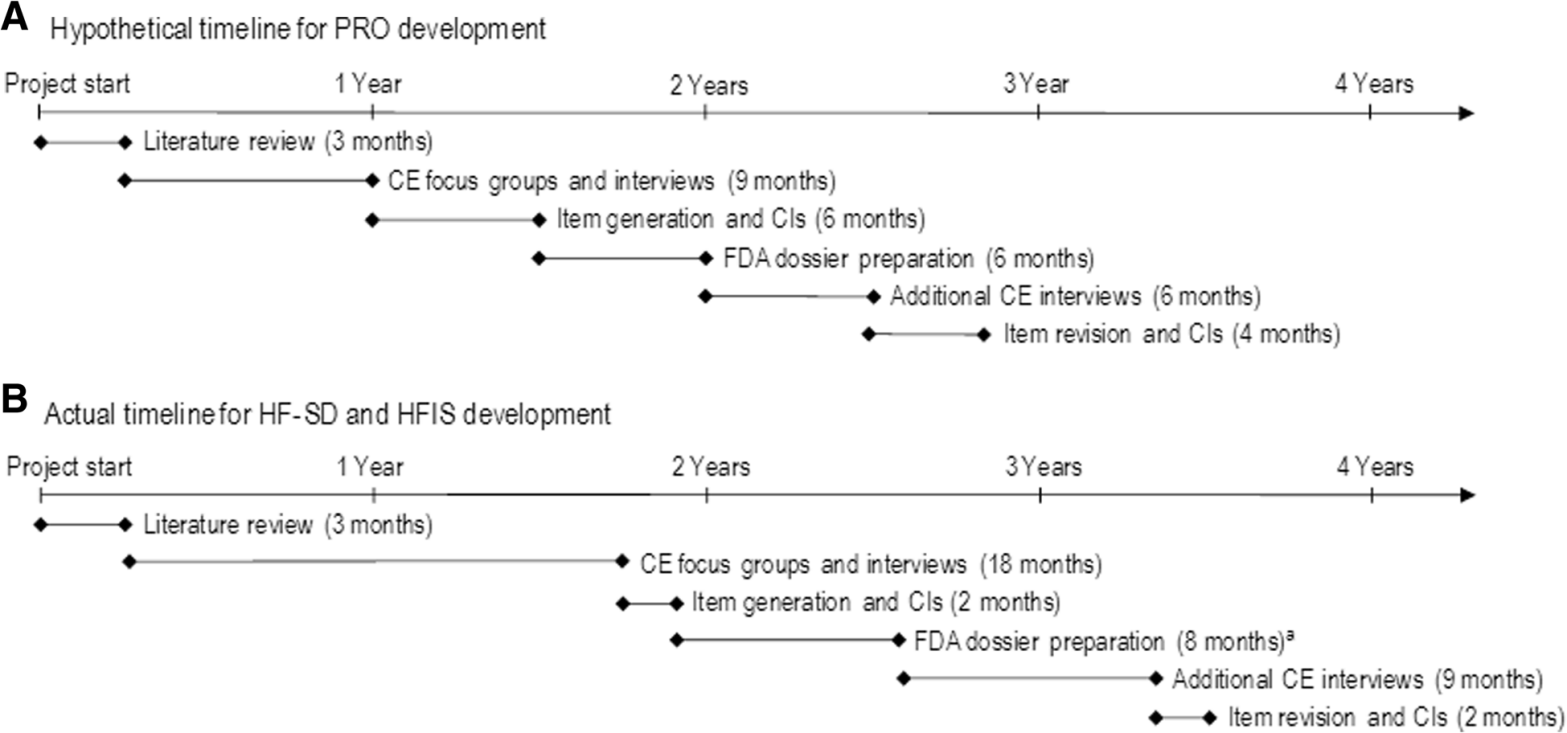
**(a)** Hypothetical and (**b**) actual timelines of PRO instrument development. ^a^Included second literature review. PRO, patient-reported outcome; CE, concept elicitation; CIs, cognitive interviews; FDA, Food and Drug Administration; HF-SD, Heart Failure Symptom Diary; HFIS, Heart Failure Impact Scale

Timelines must be carefully considered for the integration of PRO development within a clinical trial program (Fig. 2). Factors that impact timelines include: designing the study; sponsor reviews; IRB interactions (which require extra time in academic settings); vendor contracts; design and implementation of PRO instruments (both paper and electronic versions); patient recruitment; translations; and communication with regulatory agencies.

*Omecamtiv mecarbil experience: The expected and actual timelines of HF PRO development stages are presented in Fig.*2*. Early planning in our program led to successful completion of the first round of qualitative interviews and drafting of instruments before the start of phase 2. Work on the PRO strategy for the omecamtiv mecarbil clinical development program started in 2012 and was expected to be completed by 2013. Several factors caused delays in the original timeline, including the need for a second round of concept elicitation interviews and cognitive interviews, difficulties with recruitment of patients with NYHA class IV disease for qualitative interviews, and the urgency and priority status of the drug. Another factor was related to potential difficulty in identifying clinical sites that could help recruit the population of interest within the desired timeframe. Sites using local IRBs usually require lengthier contracting and review processes than sites with central IRBs.*

*Lessons learned: An early start is critical as delays in the original timeline are likely. Sponsors should anticipate and plan for a second round of qualitative studies, including strategies for recruitment of specific types of patients.*

#### Cost

The development of a new PRO instrument and the incorporation and use of the instrument in clinical trials is expensive, and the final cost is difficult to estimate. For example, in 2011 the total cost estimate of developing a new PRO instrument ranged from $725,000 to $2,150,000 [18]. Factors that significantly affect cost are shown in Table 1.

**Table 1:** Factors that affect the cost of PRO development

Factor	Considerations
Patient and site recruitment	Use of vendors to identify and recruit patients, payment for participation in qualitative studies, ease of access to target patient groups
Multiple stakeholder involvement	PRO experts, IRBs, clinicians, patients, researchers, PRO and ePRO vendors
Number of iterations	Multiple rounds of concept elicitation interviews and cognitive interviews to achieve saturation of concepts and satisfactory evidence of content validity
Delays in clinical program unrelated to PRO development	Need to update reviews prior to regulatory approval, increased risk of staff changes reducing efficiency of process
Approach selected for quantitative validation	Stand-alone vs regular clinical trials
Mode of instrument administration	ePRO vs pencil and paper vs mixed mode; need to demonstrate instrument equivalence
Clinical trial design	Number of languages and cultural adaptations needed
Dissemination strategies	Dossier preparation, conference presentations, manuscripts, etc.

*PRO* patient-reported outcome, *IRBs* Institutional Review Boards

*Omecamtiv mecarbil experience: The planning of the PRO strategy for the omecamtiv mecarbil clinical development program started in 2011 with a literature review and the initial budget for the PRO development was within the cost estimate published in 2011. The need to update the literature review prior to regulatory submission, and to conduct a second round of concept elicitation interviews and cognitive interviews and secondary collection of clinical data from the first round of interviews based on FDA feedback adversely affected the timeline and increased the cost. With careful planning we have managed to save costs by including the ePRO measure in the phase 2 clinical trial, thus eliminating the need for a stand-alone study.*

*Lessons learned: The iterative nature of qualitative studies can easily increase costs, but this can be offset by initiating ePRO development early in the planning process.*

### Engagement with various stakeholders

Many people and agencies are involved in the development of a PRO measure. Patients with the disease of interest are traditionally included as participants in concept elicitation interviews and cognitive interviews, and there is a current shift toward partnership between study sponsors and patients in research. Practicing clinicians are included in the development process to comment on clinical perspectives. In our experience, it is helpful to obtain feedback from the FDA early in PRO development.

*Omecamtiv mecarbil experience: A PRO committee was formed to oversee development of the new instruments. Patients with CHF were involved in concept elicitation and cognitive interview studies. Within the sponsor organization, representatives from Health Economics and Outcomes Research, Biostatistics, Commercial, Clinical Development, and Regulatory Affairs departments contributed to the PRO development program. Qualitative studies were designed and implemented by a vendor. FDA review and feedback was solicited. Clinician and payer feedback were solicited through market/payer research advisory boards and 1:1 interviews.*

*Lessons learned: Involvement of multiple stakeholders in PRO development ensures and improves the quality of the final measure and contributes to better understanding of the role a PRO can play in clinical trials for involved parties. Careful planning and management of the practical aspects of stakeholder involvement is critical for the success of the program.*

## Discussion and conclusions

A recent paper from Anker et al. [6] highlights the importance of integration of PRO measures in clinical trials and notes that currently PROs are not routinely included in CVD trials with the intent to support label claims. The authors advocate for an increased focus on the inclusion of scientifically sound PRO measures in CVD trials. While the Anker paper provides some broad recommendations and considerations in the design of clinical trials with the integration of a PRO, it does not provide specific details on the practical challenges and solutions for including PROs that can support labeling claims. This also applies to the recent publications by Thompson et al. and Garin, et al., which provide reviews of the use of HF PROs in HF trials [2, 19]. While existing publications describing the development and psychometric evaluation of PRO measures provide detailed theoretical steps, they rarely address the practical challenges and decision-making required in the process. Our report builds on the broad consideration from the Anker paper and provides a specific practical example of the integration process and an insight into the lessons learned.

Several governmental, nongovernmental and professional organizations have recognized the value of PROs and are actively developing or acknowledging the need to develop disease-specific PRO instruments for both regulatory approval and quality assessment. In 2005, the National Institutes of Health (NIH) established the Patient Reported Outcomes Measurement Information System (PROMIS), comprising multiple item banks built on existing instruments measuring a range of outcomes. PROMIS is currently used by various US governmental agencies (including the FDA) to assist with development and quality assessment of PRO measures [20]. In 2012, the Centers for Medicare and Medicaid Services (CMS) funded the National Quality Forum (NQF) to develop guidance on PRO measures based on the National Quality Strategy (NQS) to improve medical care for individuals [21]. The CMS now considers the patient perspective in the development of all PRO measures [20]. The Critical Path Institute (C-Path) is a nonprofit, public-private organization that partners with the FDA, PRO researchers, pharmaceutical companies, and the NIH to develop data standards, measurement standards, and methods standards to evaluate new therapies [22]. In 2013, the American Heart Association (AHA) published a scientific statement emphasizing the importance of measuring patients’ health status using PROs [23], followed by a similar endorsement by the European Society of Cardiology (ESC) a year later [6]. Other professional societies have been involved in activities to further scientific development of cardiovascular assessment measures [24, 25]. Notably, the Patient-Centered Outcomes Research Institute (PCORI) has funded an effort to develop PROMIS Condition-Specific Impact Assessment (PROMIS-CSIA) for patients with HF and osteoarthritis [26]. All PCORI-funded studies require engagement of patients as collaborators as a condition for funding.

Developing a new PRO measure in accordance with the FDA PRO guidance to support a label claim requires significant investment and early planning in the development of a clinical trial program. In this paper, a real-world example in the area of HF was provided to illustrate some of the successes and challenges experienced and to report on the methods of PRO development for HF symptom and impact measures.
